# Detection of porcine cytomegalovirus, a roseolovirus, in pig ovaries and follicular fluid: implications for somatic cells nuclear transfer, cloning and xenotransplantation

**DOI:** 10.1186/s12985-023-01975-7

**Published:** 2023-01-27

**Authors:** Sabrina Hansen, Konrad Fischer, Ludwig Krabben, Alexander Rinke Carrapeiro, Bernhard Klinger, Angelika Schnieke, Benedikt Kaufer, Joachim Denner

**Affiliations:** 1 Institute of Virology, Free University Berlin, Berlin, Germany; 2 Chair of Animal Biotechnology, TUM School of Life Sciences Weihenstephan, Technical University Munich, Freising, Germany

**Keywords:** Porcine cytomegalovirus, Porcine roseolovirus, Xenotransplantation, Oocytes, Somatic cell nuclear transfer, Cloning

## Abstract

**Background:**

Porcine cytomegalovirus (PCMV) is a porcine roseolovirus (PCMV/PRV) which is widely distributed in pigs. Transmission of PCMV/PRV in preclinical xenotransplantations was shown to significantly reduce the survival time of the pig transplants in non-human primates. PCMV/PRV was also transmitted in the first transplantation of a pig heart into a human patient. To analyze how PCMV/PRV could be introduced into pig breeds, especially considering cloned transgenic pigs, and subsequently spread in breeding facilities, we screened ovaries and derived materials which are used to perform somatic cell nuclear transfer (SCNT).

**Methods:**

DNA was isolated from ovarian tissues, follicular fluids, oocytes with cumulus cells, denuded oocytes and parthenotes. A real-time PCR with PCMV/PRV-specific primers and a probe was performed to detect PCMV/PRV. Furthermore, a Western blot assay using a recombinant fragment of the gB protein of PCMV/PRV was performed to screen for virus-specific antibodies in the follicular fluids.

**Results:**

PCMV/PRV was found by real-time PCR in ovarian tissues, in the follicular fluid and in oocytes. In parthenotes the virus could not be detected, most-likely due to the low amount of DNA used. By Western blot assay specific antibodies against PCMV/PRV were found in 19 of 20 analyzed follicular fluids.

**Conclusion:**

PCMV/PRV was found in ovarian tissues, in the follicular fluids and also in denuded oocytes, indicating that the virus is present in the animals of which the oocytes were taken from. Despite several washing steps of the denuded oocytes, which are subsequently used for microinjection or SCNT, the virus could still be detected. Therefore, the virus could infect oocytes during genetic modifications or stay attached to the surface of the oocytes, potentially infecting SCNT recipient animals.

**Supplementary Information:**

The online version contains supplementary material available at 10.1186/s12985-023-01975-7.

## Background

Porcine cytomegalovirus (PCMV) is a porcine roseolovirus (PCMV/PRV) [1], which poses a considerable risk for xenotransplantation using pig cells, tissues and organs. Xenotransplantation is one solution to alleviate the shortage of human transplants and it has achieved great success in the last years with long survival times of pig hearts, kidneys and other organs in non-human primates [2, 3]. However, PCMV/PRV transmission to the recipients drastically reduced the survival time of the xenotransplant, in the case of orthotopic heart transplantation into baboons from 195 days to less than 30 days [4], in the case of kidney transplantation into baboons from to 53 to 14 days [5] and in the case of kidney transplantation into cynomolgus monkeys from to 28 to 9 days [6]. For review and schematic presentation of the results see [7, 8]. In the baboons which received a PCMV/PRV-positive heart, an increase of certain cytokines such as interleukin 6 (IL-6) and tumor necrosis factor alpha (TNF-alpha) was observed [4]. Furthermore, high levels of tissue plasminogen activator–plasminogen activator inhibitor type 1 (tPA–PAI‑1) complexes were found in the transplanted baboons, suggesting a complete loss of the pro‑fibrinolytic properties of the endothelium [4]. The presence of PCMV/PRV was usually associated with consumptive coagulopathy (CC) [9], but when PCMV/PRV-free transplants were used, CC was significantly reduced [10]. PCMV was also transmitted by the first transplantation of a pig heart into a human patient, who survived for remarkable 2 months [11]. The clinical features observed in the patient were similar to the features seen in the baboons which received a PCMV/PRV-positive pig heart [4], suggesting that the virus may have contributed among other factors to the death of the patient.

The donor animal of the heart for the patient had 10 genetic modifications (10-GE pigs), including targeted insertion of two human complement inhibitor genes (human decay-accelerating factor, hDAF or hCD55; and human membrane cofactor protein, hCD46), two human anticoagulant genes (human thrombomodulin, hTM, and human endothelial protein C receptor, hEPCR), and two immunomodulatory genes (human signal regulatory protein alpha, hCD47, and human heme oxygenase 1, hHO1), as well as knockouts (KO) of 3 enzymes creating the porcine carbohydrate antigens: α1,3-galactosyltransferase (GGTA1), cytidine monophosphate-*N*-acetylneuraminic acid hydroxylase (CMAH), and β-1,4-*N*-acetyl-galactosaminyl transferase 2 (B4GALNT2) as well as the porcine growth hormone receptor (GHR). These 10-GE pigs do not express red blood cell antigens and are therefore universal donors with respect to blood type [11]. For the genetic modification and the cloning of the pigs, oocytes had to be used for SCNT. As each SCNT requires hundreds of oocytes, almost all laboratories use oocytes from slaughterhouse pigs. For maturation and modifications, these oocytes are cultivated in vitro, based on follicular fluid culture media in the past, which meanwhile has been replaced by fully synthetic maturation media. After having performed the desired modifications, e.g., by microinjection or SCNT, these oocytes are transferred to a surrogate mother to give birth to gene-modified piglets. We recently summarized the literature clearly showing the risk of viral transmission, including herpesviruses, by oocytes or follicular fluid in numerous cases [12]. In order to analyze, whether this may happen in the case of PCMV/PRV, ovarian tissues, oocytes with and without cumulus cells, follicular fluid and other tissues were screened for the presence of PCMV/PRV.

## Methods

### Pigs and porcine tissues

Porcine oocytes and tissues were collected at a local slaughterhouse. As this slaughterhouse receives pigs from Germany, Austria and Czech Republic, animals had several genetic backgrounds including among others Deutsche Landrasse, two-breed crossing out of Edelschwein and Pietrain, and three-breed crossing of Edelschwein, Deutscher Landrasse and Pietrain. Ovaries from this slaughterhouse have been regularly used by several groups to isolate oocytes and to perform genetic modifications. The age of the pigs was about 6 months with a weight between 100 and 120 kg.

### Isolation of oocytes

Ovaries from prepubertal gilts were isolated and transported to the laboratory at 38 °C in phosphate buffered saline (PBS) supplemented with antibiotics and antimycotics. Ovaries were rinsed several times with warm PBS, supplemented with 1% cetyl trimethyl ammonium bromide (CTAB), an antiseptic solution with various antibacterial, antifungal and antiviral properties. Subsequent washing steps were performed with warm PBS solution. Ovaries were placed in warm PBS and kept at 38 °C. Follicles with a diameter of 3–6 mm were punctured using a 10 ml syringe and a 18G needle. Porcine follicular fluid was extracted and stored at 38 °C. Oocytes with cumulus cells complexes settled at the bottom of the tube were subsequently isolated. 6–8 ml of working medium (WM), which consists of medium 199 and 10% FCS supplemented with 1% amphotericin B and 1% penicillin–streptomycin, were mixed with the cells and transferred to a petri dish for collection. High quality oocytes with dark, evenly granulated cytoplasm and several compact layers of cumulus cells were identified under a stereomicroscope. Oocytes were rinsed twice in WM to remove cell debris. Oocytes were transferred with a mouth pipette and self-made glass capillaries of about 300 µm diameter, also to make washing steps as efficient as possible.

### In vitro maturation

For in vitro maturation, oocytes were transferred to a triple gas incubator (5% O_2_, 5% CO_2_, 90% N_2_, set to 38.5 °C humidified atmosphere). Oocytes were rinsed with maturation medium and transferred to a separate maturation well. After 45 h, successful maturation was confirmed by visual assessment of polar body extrusion from a sample group of ovaries. Maturation was carried out in a chemically defined maturation medium which consists of 500 ml medium 199, 27.5 mg glucose, 5 mg sodium pyruvate, 0.5 mL penicillin–streptomycin and 1 mL 3% polyvinyl alcohol.

### Denuding and parthenogenesis

Mature oocytes were denuded in WM supplemented with 1 mg/ml hyaluronidase and rinsed twice in working medium. They were controlled for granulated cytoplasm and extrusion of the first polar body. Chemical activation was conducted in WM supplemented with 25 µm ionomycin (calcium ionophore) for 10 min. Oocytes were washed twice in WM and once in PZM5, a defined medium for embryos, and subsequently placed in the incubator in 500 µl of PZM5 supplemented with 5 µg/ml of cytochalasin for 3 h. Afterwards they were rinsed twice in working medium and once in PZM5. Parthenotes were used for our assays to increase the extractable DNA amount from single oocytes and to avoid possible PCMV/PRV viral transfer by sperm.

### Sample storage

Samples of porcine ovaries and follicular fluids were frozen and stored at − 20 °C. Oocytes with cumulus cells, denuded oocytes and parthenotes were collected in PBS and stored at − 20 °C.

### DNA extraction

DNA was isolated from ovarian tissues (20 mg), follicular fluid (200–400 µl), maturation medium (200 µl), oocytes with or without cumulus cells (5–250 oocytes per sample) and parthenotes (5–15 cells per sample) using the innuPREP Virus DNA/RNA Kit (Analytik Jena, Jena, Germany). The DNA/RNA was eluated in 30 or 60 µl nuclease-free water. Samples were stored at − 20 °C until further processing.

### Real-time polymerase chain reaction (PCR)

The detection of PCMV/PRV was performed using a real-time PCR assay with specific primers and a probe developed by Mueller et al. [13] (Table 1). All assays were performed as duplex real-time PCR using as reference gene porcine glyceraldehyde-3-phosphate-dehydrogenase (pGAPDH) with a specific primer–probe mixture (Table 1) [14] running 45 cycles as described previously [15–17]. All experiments were performed with the SensiFAST Probe No-ROX kit (Meridian Bioscience, Cincinnati, OH, USA) and the qTOWER3 G qPCR cycler (Analytik Jena, Jena, Germany). A reaction volume of 20 µl was prepared containing 1.8 µl of PCMV/PRV-FAM mix with 1.8 µl of pGAPDH-HEX mix as internal control and 4.0 µl of extracted DNA. The reaction for the PCMV/PRV real-time PCR was carried out for 2 min at 50 °C for activation, 10 min at 95 °C followed by 45 cycles comprising 15 s at 95 °C for denaturation and 60 s at 60 °C for annealing and elongation. As positive control a PCMV/PRV-specific gene block, as negative control water or medium were used as described [15].

**Table 1:** Primers and probes used in this study

PCR assay	Accession number	Primer/probe	Sequence 5′–3′	Location (nucleotid number)	References
PCMV*	AF268040.2	PCMV-Fwd	GTT CTG GGA TTC CGA GGT TG	5074–5093	[13, 15–17]
		PCMV-Rev	ACT TCG TCG CAG CTC ATC TGA	5036–5116	
		PCMV-Probe	6FAM-CAG GGC GGC GGT CGA GCT C-BHQ	5095–5113	
pGAPDH	NM_001206359.1	pGAPDH-Fwd	ACA TGG CCT CCA AGG AGT AAG A	1083–1104	[14, 46]
		pGAPDH-Rev	GAT CGA GTT GGG GCT GTG ACT	1188–1168	
		pGAPDH-Probe	HEX-CCA CCA ACC CCA GCA AGA GCA CGC-BHQ	1114–1137	

*PCR* Polymerase chain reaction, *PCMV* Porcine cytomegalovirus, *pGAPDH* Porcine glyceraldehyde-3-phosphate dehydrogenase, *Fwd* forward primer, *Rev* Reverse primer, *nt* nucleotide, *6FAM* 6-carboxyfluorescein, *BHQ* Black hole quencher, *HEX* hexachlorofluorescein

*The PCMV real-time PCR was modified and performed as duplex PCR.

### Western blot analysis

Western blot analysis was performed as previously described [16, 18]. Briefly, for the detection of antibodies against PCMV/PRV, the Western Blot assay designed by Plotzki et al. [18] was re-established, but only the C-terminal fragment R2 of the gB protein of PCRV/PRV was used as antigen because the R2 protein was shown to be immunodominant [18]. The R2 fragment of the gB of PCMV/PRV was produced in E. coli BL21 cells using the pET16b expression vector encoding PCMV-R2 as described in detail [16, 18]. Cell were induced with 1 mM isopropyl-β-D-thiogalactopyranosid (Roth, Karlsruhe, Germany), harvested, and dissolved in 10 mL 8 M urea, 0.5 M NaCl, 15 mM imidazole, 20 mM Tris pH 7.5. The supernatant after centrifugation was applied to a HisTrap HP column installed on a Äkta Prime Plus system (both GE Healthcare, Chicago, Illinois, USA), and eluted after washing using 6 M urea, 0.5 M NaCl, 500 mM imidazole, 20 mM Tris pH 7.5. The purified R2 protein was characterized by a sodium dodecylsulfate polyacrylamide gel electrophoresis (SDS-PAGE) using 12% gels and to obtain sharper bands 17% gels. The protein was dissolved in sample buffer (375 mM Tris–HCl, 60% glycerol, 12% SDS, 0,6 M DTT, 0.06% bromophenol blue) and denatured for 5 min at 95 °C prior to electrophoresis. The SDS PAGE was run in a Mini-Protean Tetra Vertical Electrophoresis Cell (Bio-Rad Laboratories, Incs., Hercules, CA, USA) using a 17% polyacrylamide gel and the PageRuler prestained protein ladder (Thermo Fisher Scientific, Waltham, USA). The protein was transferred for 100 min to a polyvinylidene fluoride membrane (ROTI PVDF, 8989.1, Roth, Karlsruhe, Germany) by electroblotting (100 mA) using the electroblotting device of peqlab Biotechnologie GmbH. After electroblotting the membrane was blocked for 1 h at 4 °C in 5% non-fat dry milk (Roth, Karlsruhe, Germany) in PBS with 0.05% Tween 20 (Roth, Karlsruhe, Germany) (PBS-T) (blocking buffer). The membrane was cut into strips and incubated over night at 4 °C with sera diluted 1:150 in blocking buffer. Afterwards, washing was performed with 0.05% PBS-T three times for 10 min each. Polyclonal goat anti-pig immunoglobulin G (IgG) Fc Secondary Antibody HRP (Invitrogen by Thermo Fisher Scientific, Waltham, USA) was diluted 1:15.000 in blocking buffer and strips were incubated for 1 h at room temperature, followed by three washing steps for 10 min each. Detection of the signal following incubation with the ECL Western Blotting Substrate (Cytiva, Amersham) was done with the FUSION-SL 3500 WL imaging device (peqlab Biotechnologie GmbH).

## Results

### Detection of PCMV/PRV in different tissues by RT-PCR

Ovarian tissues, follicular fluid, and ocytes with or without cumulus cells were collected from up to 20 slaughterhouse animals. In addition, parthenotes were obtained as described in Methods. As negative control, a commercial maturation medium was analyzed, which is used for the cultivation of oocytes instead of a medium containing follicular fluid. DNA was isolated and a duplex real-time PCR was performed using PCMV/PRV-specific primers and a probe. It is important to note that the target sequence of the real-time PCR is a sequence in the polymerase gene which is highly conserved among all PCMV/PRV. Primers and a probe specific for porcine GAPDH were used to control the presence of porcine DNA. Three samples out of 20 samples from 20 animals were PCMV/PRV-positive when ovarian tissues were analyzed (Table 2). The results of the GAPDH testing showed that sufficient DNA was present in all samples and that the DNA input was identical in all samples (Table 2, Additional file 1: Figure S1). Two out of 20 samples of follicular fluid were positive. 40 oocytes with cumulus cells from eight samples, 40 denuded oocytes from 8 samples and 30 parthenotes from 6 samples were negative in this assay. Parthenotes are oocytes that can be activated, among others, by high calcium concentrations or electric stimuli. As no sperm is required for oocyte activation, this excludes the risk of sperm-mediated PCMV/PRV transmission. Due to the higher cell number of parthenotes compared to oocytes, the DNA amount that can be obtained is much higher for subsequent PCR analysis.

**Table 2:** Results of the real-time PCR testing

Tested material	Number PCMV/PRV-positive/number tested	Ct values of the PCMV/PRV-positive samples	Ct values of all pGAPDH-positive samples, median ± SD
Ovarian tissue	3/20	34.6; 36.2; 38.1	20.5 ± 1.0
Follicular fluid	2/20	33.4; 33.7	17.8 ± 1.3
Oocytes with cumulus cells	0/6	No ct	28.7 ± 1.7
Denuded oocytes	0/8	No ct	35.7 ± 1.1
Parthenotes	0/6	No ct	37.4 ± 0.6
Maturation medium	0/3	No ct	No ct

Most importantly, when higher amounts of oocyte DNA (50 ng DNA per PCMV/PRV real-time PCR in triplicates) from 100 pooled oocytes was used, a positive reaction was found with the oocytes, as well as in 50 ng DNA per PCMV/PRV real-time PCR from 150 pooled oocytes, indicating that PCMV/PRV is present in or on oocytes despite the several washing steps (Table 3) (Additional file 1: Figure S1). As expected, the control maturation medium was negative (Table 2).

**Table 3:** Results of the extended real-time PCR testing of oocytes

Tested material	Number of tested cells	Ct values of the PCMV/PRV-positive samples	Ct values of all pGAPDH-positive samples, median ± SD
Oocytes with cumulus cells	40	No ct	28.7 ± 1.7
Denuded oocytes	40	No ct	35.7 ± 1.1
Parthenotes	30	No ct	37.4 ± 0.6
100 oocytes pooled	100	35.9	27.6
150 oocytes pooled	150	36.9	26.0

### Detection of antibodies against PCMV/PRV by Western blot

**Fig. 1:**
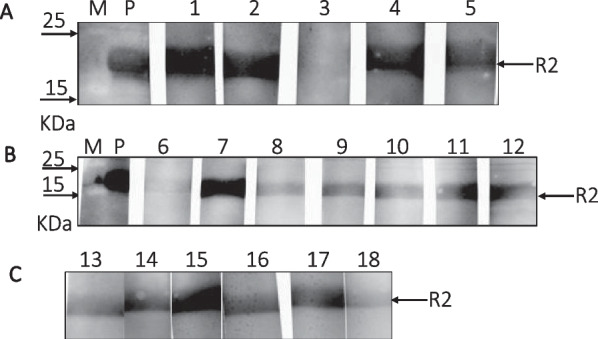
Western blot analysis of 18 follicular fluids (1–18). **A** a 12% gel was used for SDS-PAGE, **B**, **C** a 17% gel was used. The molecular weight marker (M), a serum from a positive control animal (P) and the location of the recombinant R2 protein of the gB protein of PCMV/PRV are indicated. The serum dilution was 1:150

In order to analyze whether antibodies against the PCMV/PRV can be found in the follicular fluid, a Western blot assay was performed. As antigen the C-terminal R2 fragment of the gB protein of PCMV/PRV was used [18]. The R2 fragment was found to be immunodominant: In comparison with the N-terminal R1 fragment more sera were detected positive [18]. 19 of 20 samples of follicular fluid were positive in this assay (Fig. 1), indicating that these animals were infected by PCMV/PRV as shown by the positive antibody reaction. The antibodies found in the follicular fluid were either present in the follicular fluid or coming from contaminating blood when collecting the follicular fluid. The latter is unlikely because the follicular fluid was carefully collected avoiding blood contaminations.

## Discussion

For the generation of genetically modified pigs, usually SCNT is the method of choice, as prescreening of modified cells is possible, thereby strongly reducing the number of incorrectly modified pigs. We showed here that in PCMV/PRV positive pigs from slaughterhouses, the ovarian tissue and oocytes are infected with the virus and that PCMV/PRV is also present in the follicular fluid. Although RT-PCR analysis only could detect PCMV/PRV in a limited number of samples, almost all samples turned out to be positive for antibodies against PCMV/PRV detected by Western blot analysis. As oocytes have initially to be isolated from the follicles together with follicular fluid and all isolations are regularly pooled, even only one PCMV/PRV positive pig could potentially infect all other oocytes during or after isolation.

This data indicates that PCMV/PRV is able to infect piglets created by SCNT and this may explain how animals got infected even in previously virus-negative facilities. Usually infection with PCMV happens early in live of the piglets by contact with the infected mother [19, 20]. PCMV is found in most tissues but mainly in the nose of newly infected newborn piglets where it causes rhinitis, in addition it also causes conjunctivitis in the eyes. Most infections are sub-clinical, clinical disease is rare. Clinical signs are only seen if PCMV infects a sow for the first time when she is late in pregnancy. In this case, fetal deaths, mummified fetuses, stillbirths, and weak piglets are observed. The virus is shed from the nose and eyes, by urine and farrowing fluids, and semen of the young pig. In experimental settings, but not under natural conditions, the virus was shown to cross the placenta [21]. Therefore, the presence of PCMV/PRV in ovarian tissue and in the follicular fluid is not surprising. The fact that oocytes are virus-positive is of importance. The oocytes are usually surrounded by the zona pellucida (ZP), an extracellular matrix of glycoproteins building a three-dimensional structure which should prevent virus infection [22]. It is widely recognized that a virus infection is only possible after damaging the ZP. After infection of ZP-damaged pig oocyte with porcine circovirus 2 (PCV2) infected embryos were detected [23]. However, transmission of viruses was also observed in the presence of an intact ZP (for review see [12]). The fact that PCMV/PRV was still found in oocytes despite several washing steps indicates that these steps are not sufficient to remove the virus, either because it is already inside the oocyte or firmly attached to the glycoproteins of the ZP. Additional decontamination techniques, for example treatment with trypsin, were also not successful in the case of some viruses such as the porcine reproductive and respiratory syndrome virus (PRRSV), the porcine parvovirus (PPV), the porcine circovirus 2 (PCV2) and the encephalomyocarditis (EMCV) [24]. Treatment with hyaluronidase was more successful [24]. Since antiviral drugs effective against human cytomegalovirus such as ganciclovir are not so effective against PCMV/PRV [25], because it is a roseolovirus [1], treatment with these toxic substances is not recommended.

Although meanwhile synthetic maturation mediums are used, previously, when follicular fluid was added to the medium, the presence of PCMV/PRV in the follicular fluid as shown here could lead to an infection of the oocytes, especially after SCNT when the ZP is disrupted.

To use pigs from slaughterhouses as source of oocytes has many disadvantages. In addition to the high prevalence of PCMV/PRV [18], numerous other viruses were found in slaughterhouse pigs, among them hepatitis E virus (HEV) [26–28], parvoviruses [29], Torque teno sus virus [30], porcine lymphotropic herpes viruses [31] and circoviruses [32]. Whether these viruses (with exeption of HEV, which is a well-known zoonotic virus (for review see [33])) pose a risk for xenotransplantation and whether they can be transmitted via SCNT is unclear. However, currently the use of slaughterhouse pigs is the only feasible solution to obtain the high number of oocytes which are regularly required to perform SCNT.

PCMV/PRV was transmitted during the first pig heart transplantation to a patient in Baltimore [11]. Since the supplier of the 10-GE pig used for this transplantation, Revivicor, supplies cloned animals, it cannot be excluded that the origin of the virus was in oocytes used for cloning. PCMV/PRV is a latent herpesvirus [34] and for the detection of latent viruses special detection strategies are needed [16]. When these detection strategies will be applied and techniques of elimination of the virus from infected herds such as early weaning will be used [35], xenotransplantation could even be safer [36, 37].

## Limitations of the study

To detect PCMV in our study, we used a modified real-time PCR originally developed by Mueller et al. [13], which was modified by us into a duplex real-time PCR. This PCR detects a conserved region in the polymerase gene of PCMV and was used by many laboratories, including ours [4, 13, 14, 16, 38, 39]. To improve the diagnostic, an additional PCR would be useful, either detecting another sequence in the polymerase genes as described by us [17, 40], or detecting a sequence in another gene, e.g., in the gene encoding the gB protein. This gene also contains regions which are conserved among all PCMV [41] and therefore can be used for the detection of the virus. The gB protein of PCMV/PRV contains even sequences which are related to HHV-6 to an extend that a cross-reactivity of antibodies was observed [42].

Another limitation of the study is that we did not investigate the presence of viral mRNA. In a productive infection there should be more mRNA than viral DNA and therefore mRNA could have been detected if the virus is expressed.

For the antibody testing we used a linear recombinant protein. Although recombinant proteins and even peptides are generally used for screening for virus infections, for example commercial tests for the detection of a HIV-1 infection [43, 44], the use of a properly folded protein may be more meaningful. When we used two recombinant fragments of the gB protein of PCMV/PRV, the N-terminal R1 and the C-terminal R2, we found that sera from most infected pigs recognized R2 [18]. Therefore, in later investigations, including this study, only R2 was used for testing [10, 16, 38].

Furthermore, it is unclear whether PCMV/PRV is replicating in the oocytes or whether it established latency. At present it remains unclear in which cells, tissues and organs PCMV/PRV establishes latency in pigs. When studying infected animals, the virus was found in different organs and the organ with the highest virus load was different in different animals [45]. The presence of PCMV/PRV sequences in different organs may be due to the presence of infected lymphocytes where the virus is replicating and also may establish latency.

## Conclusion

The presence of PCMV/PRV in ovarian tissue, oocytes and follicular fluid of PCMV/PRV-positive pigs indicates that using oocytes and follicular fluid from these animals for SCNT may lead to PCMV/PRV-positive piglets. Therefore, it is advised to use PCMCV/PRV-negative animals as oocyte donors or to use effective decontamination techniques.

## Supplementary Information

**Additional file 1:** Demonstration of real-time PCR testing of pig oocytes for PCMV/PRV. As positive control a gene block was used.

## Data Availability

All data are part of this manuscript.

## Abbreviations

B4GALNT2: β-1,4-*N*-acetyl-galactosaminyl transferase 2
CC: Consumptive coagulopathy
CMAH: Cytidine monophosphate-*N*-acetylneuraminic acid hydroxylase
CTAB: Cetyl trimethyl ammonium bromide
GHR: Growth hormone receptor
GGTA1: α1,3-Galactosyltransferase
hDAF: Human decay-accelerating factor
hEPCR: Human endothelial protein C receptor
hCD46: Human membrane cofactor protein
hTM: Human thrombomodulin
hEPCR: Human endothelial protein C receptor
hCD47: Human signal regulatory protein alpha
hHO1: Human heme oxygenase 1
IL-6: Interleukin 6
PCMV/PRV: Porcine cytomegalovirus/porcine roseolovirus
pGAPDH: Porcine glyceraldehyde-3-phosphat3-dehydrogenase
SCNT: Somatic cell nuclear transfer
TNF-alpha: Tumor necrosis factor alpha
tPA‑PAI‑1: Tissue plasminogen activator-plasminogen activator inhibitor type 1 WM working medium
