# Supplementary material for: Detection of porcine cytomegalovirus, a roseolovirus, in pig ovaries and follicular fluid: implications for somatic cells nuclear transfer, cloning and xenotransplantation
Source: Virol J. 2023 Jan 27;20:15. doi: 10.1186/s12985-023-01975-7 (PMC9881377; doi:10.1186/s12985-023-01975-7)
Supplement: Supplementary file 1 — Additional file 1: Demonstration of real-time PCR testing of pig oocytes for PCMV/PRV. As positive control a gene block was used. [file 12985_2023_1975_MOESM1_ESM.pptx]

## Slide 1
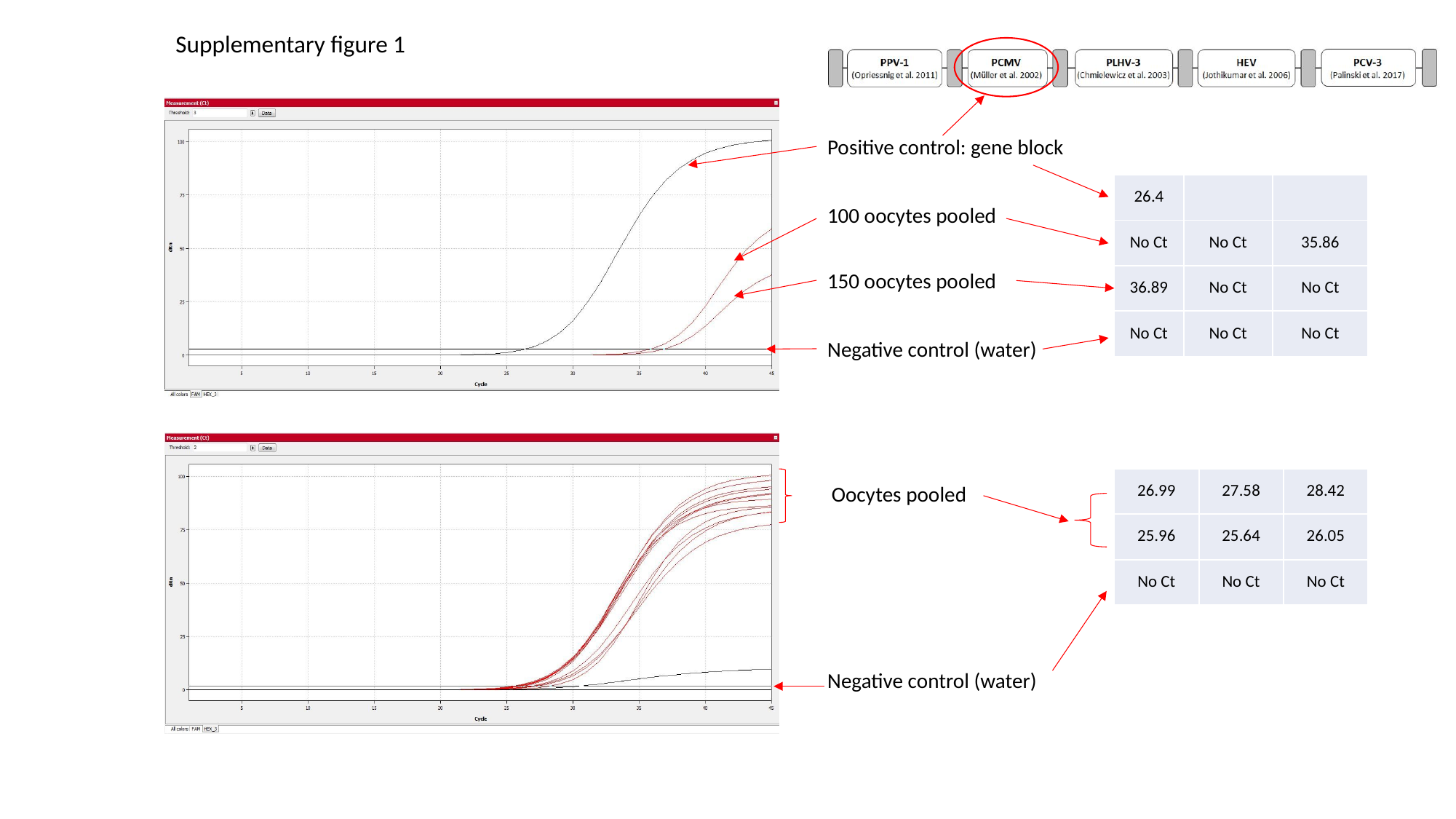

Supplementary figure 1
Positive control: gene block
| 26.4 | | |
| --- | --- | --- |
| No Ct | No Ct | 35.86 |
| 36.89 | No Ct | No Ct |
| No Ct | No Ct | No Ct |
100 oocytes pooled
150 oocytes pooled
Negative control (water)
| 26.99 | 27.58 | 28.42 |
| --- | --- | --- |
| 25.96 | 25.64 | 26.05 |
| No Ct | No Ct | No Ct |
Oocytes pooled
Negative control (water)
